# Invasive liver abscess syndrome caused by *Klebsiella pneumoniae*: first Tanzanian experience

**DOI:** 10.11604/pamj.2020.36.191.23070

**Published:** 2020-07-16

**Authors:** Nadeem Mehboob Kassam, Omar Mohamed Aziz, Samina Sadrudin Somji, Zainab Yusuf Fidaali, Salim Ramzan Surani

**Affiliations:** 1 Department of Internal Medicine, Aga Khan University Medical College, Nairobi, Kenya, East Africa,; 2 Department of Internal Medicine, Aga Khan Hospital, Dar es Salaam, Tanzania,; 3 Department of Radiology, Aga Khan Hospital, Dar es Salaam, Tanzania,; 4 Internal Medicine, Texas A&M Health Science Center, Bryan, United States of America

**Keywords:** Hepatic abscess, invasive syndrome, *Klebsiella pneumoniae*

## Abstract

Over the past 20 years there has been growing awareness of community-acquired primary liver abscess caused by strains of Klebsiella pneumoniae (K. pneumoniae) especially in patients of Asian descent, a minority of which are characterized by metastatic spread. A common and frequent destructive complication is endophthalmitis as well as the involvement of the central nervous system (CNS), causing suppurative meningitis or brain abscess. Here we report a case of invasive liver abscess caused by K. pneumoniae in an Asian patient who presented to our hospital in Tanzania with bilateral lower limb swelling for 6 weeks with acute onset of difficulty in breathing.

## Introduction

*K. pneumoniae* is a gram-negative member of the *Enterobacteriaceae* family which has the ability to cause infection at a variety of sites. Recently a distinctive clinical syndrome has developed globally which is characterized by liver abscesses and associated with metastatic complications. This syndrome is believed to be community-acquired, especially in people of Asian descent [1]. Detection of specific capsular serotypes (K1 and K2) and the presence of a specific gene (magA and rmpA genes) in K. pneumoniae have been associated with this invasive syndrome [2]. The diagnosis of Klebsiella Liver Abscess (KLA) is established when *K. pneumonia* is cultured either from blood or the aspirate of the abscess in the absence of underlying hepatobiliary disease.

## Patient and observation

A 45-year old man of Philippine-origin was admitted with a six-week history of progressive bilateral lower limb swelling associated with aches and occasional cramps while walking. He reported unintentional weight loss of about 10 kilograms (kg) in the past 3 months with a two-week history of intermittent fevers and one-day history of sudden onset of difficulty breathing which was initially only on exertion but on hospitalization was present at rest. This was not accompanied by cough or chest pain. He had been treated with several courses of oral antibiotics with little clinical improvement. The patient was known to be hypertensive for the past 5 years on daily Losartan 50 mg and Amlodipine 10 mg. He worked on a cruise ship as a welder for many years. On general examination he was found to be alert, dyspneic (RR = 30 cycles/min), desaturating to 89% on room air, and febrile (38.2°C) with a heart rate of 112 beats/min. The patient was moderately pale with scleral jaundice and bilateral pitting lower limb edema; an erythematous patch on the anterior aspect of the right leg was noted which measured around 4 cm x 3 cm that was warm and tender to touch. On respiratory system examination the patient had a normal chest cage with the trachea centrally located. Fine crepitations were auscultated bilaterally from the midzone to bases. On abdominal examination there was uniform distension with an everted umbilicus and mild tenderness was elicited on deep palpation of the right hypochondrium. The liver span was 12 cm and the spleen was not palpable. Initial blood work done revealed a markedly elevated white count of 31.4 K/μL, anemia with a hemoglobin (Hb) of 6.0 g/dL, an elevated C-Reactive Protein (CRP) of 221.48 mg/dL, Procalcitonin (PCT) of 61 ng/mL and D-Dimer of 6.74 μg/mL. He had an elevated direct bilirubin of 23.32 μmol/L but otherwise normal liver and renal function. Erythrocyte sedimentation rate (ESR) was 30 mm/hr. HIV was negative, as was blood serology and stool microscopy for Amoeba.

**Figure 1:**
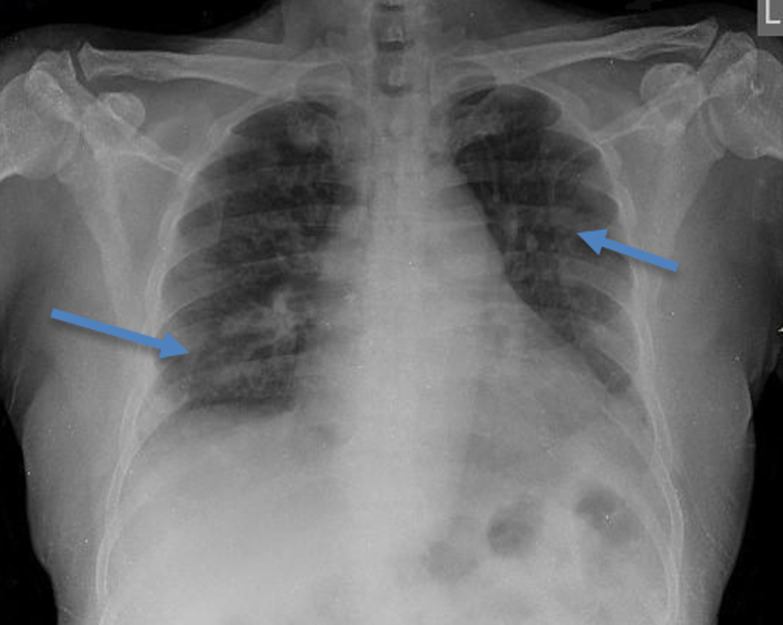
chest X-ray, bilateral parenchymal infiltrates likely of infective etiology

**Figure 2:**
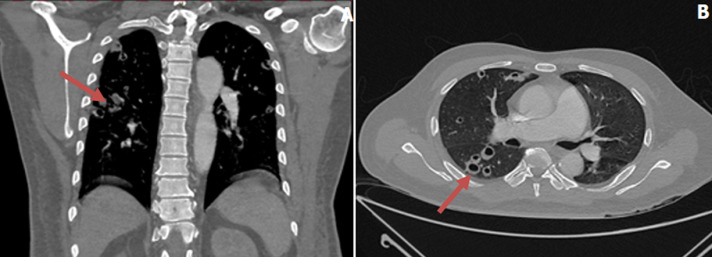
A, B) CT chest (coronal and axial views): multiple parenchymal and pulmonary nodular lesions, some with air-fluid levels (abscesses), indicated by blue arrows

**Figure 3:**
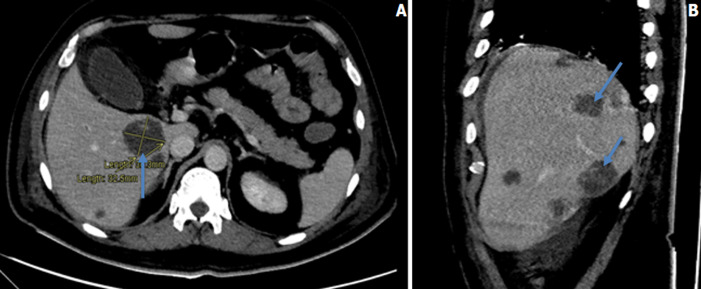
A, B) CT-abdomen: multiple non-enhancing cystic liver lesions with largest measuring 3.6 x 3.2 cm

**Figure 4:**
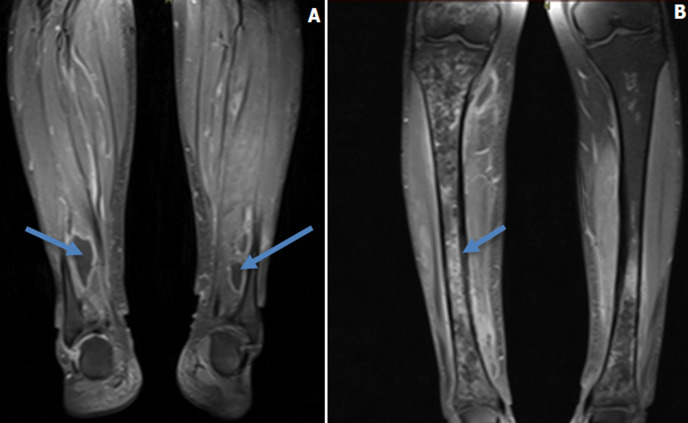
A) post-contrast T1 fat-suppressed MRI image showing bilateral subcutaneous and intramuscular multiple small collections; B) post-contrast T1 fat-suppressed MRI shows subtle enhancement of bone marrow with no cortical break, elevation nor obvious periostitis suggestive of osteomyelitis

An initial chest x-ray revealed parenchymal infiltrates (Figure 1) likely of infective etiology and subsequent radiological workup showed metastatic abscesses in the lungs, liver, lower limb muscle and subcutaneous tissue as well as bone marrow changes suggestive of osteomyelitis. Computed tomography (CT) scan of the abdomen showed multiple non-enhancing cystic liver lesions with largest measuring 3.6 x 3.2 cm (Figure 2 A,B, Figure 3 A,B, Figure 4 A,B). Doppler ultrasound for both limbs were suggestive of superficial as well as deep vein thrombosis. CT-pulmonary angiography revealed no evidence of pulmonary embolism. CT of the brain showed only mild cortical atrophy. The patient was admitted to the intensive care unit and was placed on 10L/min of supplemental oxygen with a non-rebreather mask and started on broad-spectrum antibiotics. The initial antibiotic regimen included intravenous (IV) Imipenem with Cilastatin and Metronidazole. He was also empirically started on antifungal therapy on the suspicion of a disseminated fungal infection however, after the first dose of Amphotericin-B he developed an anaphylactic response and this was therefore discontinued. For deep vein thrombosis, he was initially started on therapeutic doses of subcutaneous Enoxaparin and then switched to oral Rivaroxaban. During his stay the patient reported complete loss of vision in the left eye. Fundoscopy and optical coherence tomography revealed features suggestive endogenous endophthalmitis. He also developed acute worsening of his breathlessness which was attributed to an NSTEMI based on elevated cardiac markers. Coronary angiography was essentially normal, while 2D echocardiography revealed septal hypertrophy with no regional wall motion abnormality. Blood cultures eventually grew Klebsiella pneumoniae which was sensitive to Ceftriaxone. He was given intravitreal Vancomycin 1000 mg and continued on oral ciprofloxacin for endophthalmitis. Gradually the patient improved, with resolution of fevers and difficulty breathing with significant drop in his infectious markers. After a 25 day stay in the hospital, he was discharged home and to continue follow up in his native country. On discharge we recommended IV ceftriaxone for another 4 weeks or until radiological resolution of the abscesses was detected.

## Discussion

The mounting incidence of complicated metastatic *K. pneumoniae* in healthy individuals is alarming [3]. The increasing number of cases from other geographical areas signify that this may progress into a universal problem [4]. It has been hypothesized that the prevalence of this disease in people of Asian ethnicity is due to genetic predisposition. There is a strong association between diabetes mellitus and KLA; the reported prevalence of KLA amongst diabetics is considered to be as high as 78% [5]. These patients are at high risk of developing advanced metastatic complications; the most common being endophthalmitis [6]. Treatment of KLA involves parenteral antibiotic therapy in addition to drainage (percutaneous drainage guided by imaging is preferred over surgical). Generally, KLA isolates are susceptible to cephalosporins [7], however, the choice of antibiotic should be tailored to the sensitivity of cultures. Antibiotic therapy is generally prescribed for four to six weeks. A prolonged duration of treatment should be considered for patients who do not show clinical improvement and continue to have persistent radiographic evidence of abscesses. Ideally treatment should continue until there is radiological resolution.

## Conclusion

This is, to our knowledge, the first published report of a patient with metastatic invasive *K. pneumoniae* in Tanzania. Better awareness and understanding about this disease by physicians will be valuable for earlier detection and initiation of evidence-based treatment.

## References

[1] Chang FY, Chou MY, Fan RL, Shaio MF (1988). A clinical study of Klebsiella liver abscess. Taiwan Yi Xue Hui Za Zhi.

[2] Turton JF, Perry C, Elgohari S, Hampton CV (2010). PCR characterization and typing of Klebsiella pneumoniae using capsular type-specific, variable number tandem repeat and virulence gene targets. J Med Microbiol.

[3] Lederman ER, Crum NF (2005). Pyogenic liver abscess with a focus on Klebsiella pneumoniae as a primary pathogen: an emerging disease with unique clinical characteristics. Am J Gastroenterol.

[4] Liew KV, Lau TC, Ho CH, Cheng TK, Ong YS, Chia SC (2000). Pyogenic liver abscess: a tropical centre's experience in management with review of current literature. Singapore Med J.

[5] Cheng DL, Liu YC, Yen MY, Liu CY, Wang RS (1991). Septic metastatic lesions of pyogenic liver abscess: their association with Klebsiella pneumoniae bacteremia in diabetic patients. Arch Intern Med.

[6] Sridhar J, Flynn HW, Kuriyan AE, Dubovy S, Miller D (2014). Endophthalmitis caused by Klebsiella species. Retina.

[7] Kang CI, Kim SH, Bang JW, Kim HB, Kim NJ, Kim EC (2006). Community-acquired versus nosocomial Klebsiella pneumoniae bacteremia: clinical features, treatment outcomes, and clinical implication of antimicrobial resistance. J Korean Med Sci.

